# Supplementary figures and images for: Signatures of Discriminative Copy Number Aberrations in 31 Cancer Subtypes
Source: Front Genet. 2021 May 13;12:654887. doi: 10.3389/fgene.2021.654887 (PMC8155688; doi:10.3389/fgene.2021.654887)

# Skin Melanoma: 8720/3, 8721/3, 8730/3

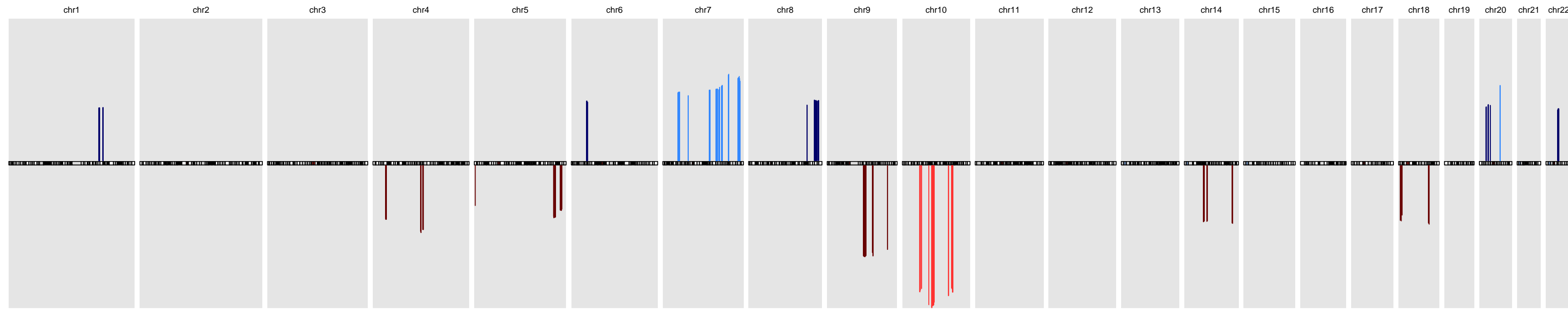

Supplement: Supplementary file 3 [file Data_Sheet_3.ZIP › signatures/Skin/Melanoma/sigGenes_full.pdf]

# Prostate Adenocarcinoma 8140/3

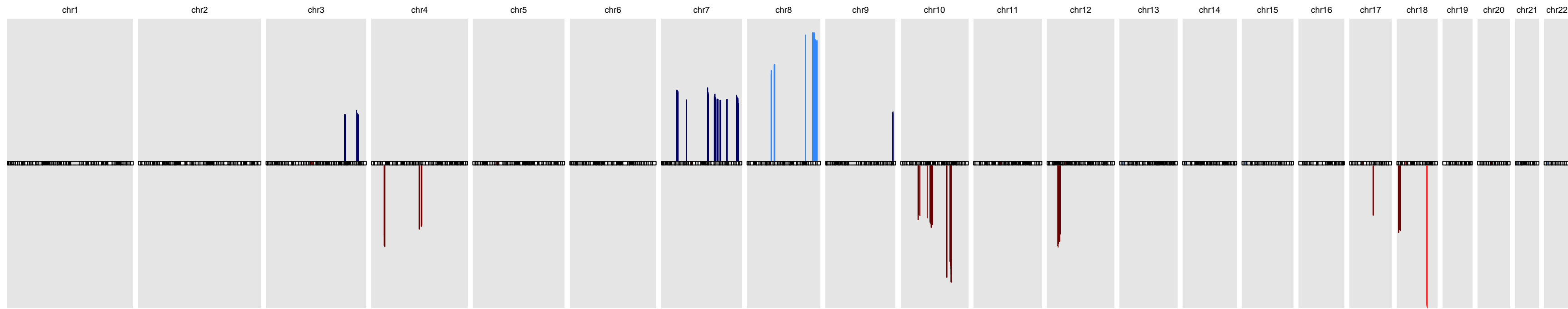

Supplement: Supplementary file 3 [file Data_Sheet_3.ZIP › signatures/Prostate/Adenocarcinoma/sigGenes_full.pdf]

# Lung Carcinoma 8010/3, 8012/3

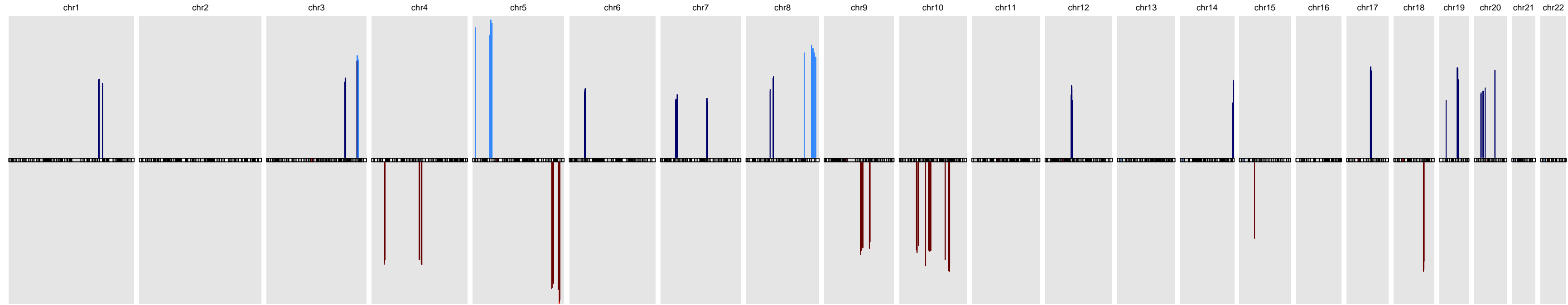

Supplement: Supplementary file 3 [file Data_Sheet_3.ZIP › signatures/Lung/Carcinoma/sigGenes_full.pdf]

# Lung Adenocarcinoma 8140/3 8255/3

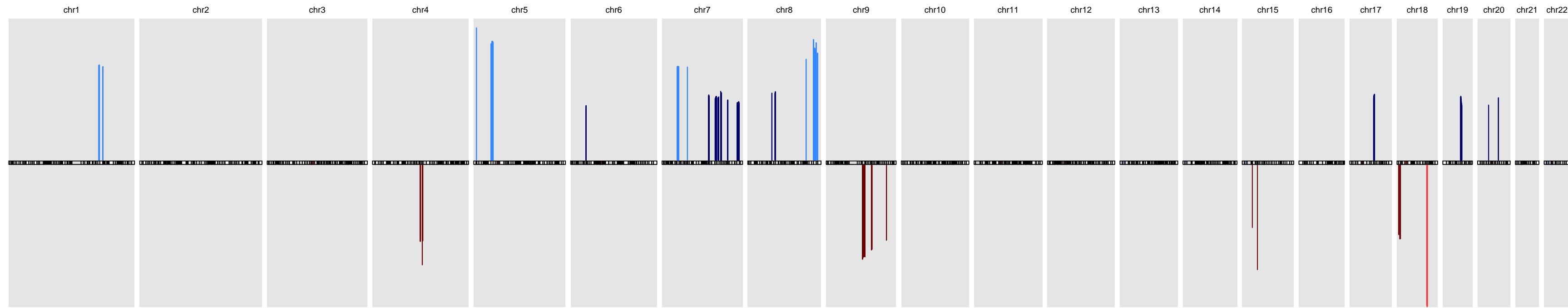

Supplement: Supplementary file 3 [file Data_Sheet_3.ZIP › signatures/Lung/Adenocarcinoma/sigGenes_full.pdf]

## Lung Small cell carcinoma 8041/3

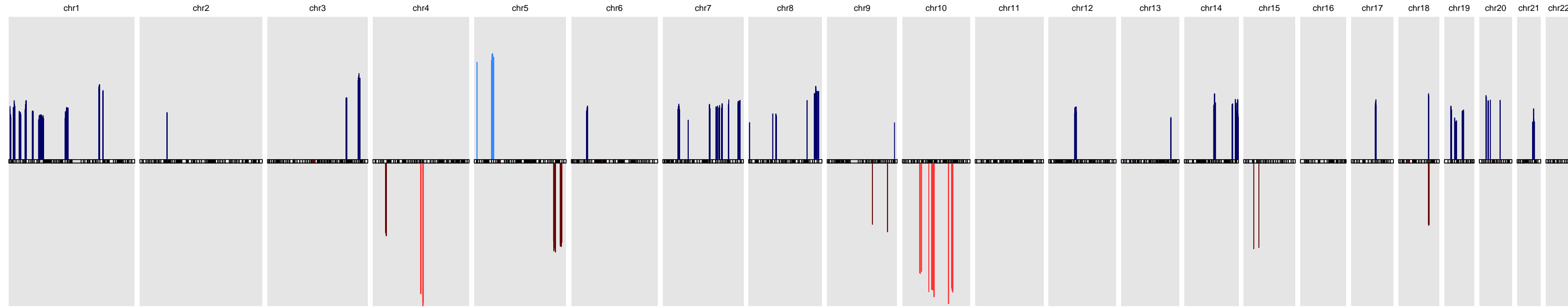

Supplement: Supplementary file 3 [file Data_Sheet_3.ZIP › signatures/Lung/Small cell carcinoma/sigGenes_full.pdf]

# Lung Squamous cell carcinoma 8070/3

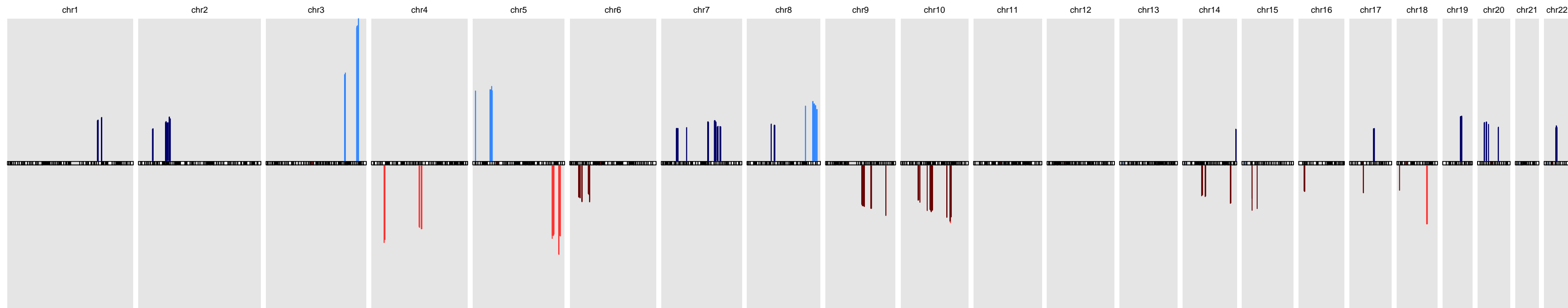

Supplement: Supplementary file 3 [file Data_Sheet_3.ZIP › signatures/Lung/Squamous cell carcinoma/sigGenes_full.pdf]

# Breast Infiltrating duct carcinoma: 8500/3

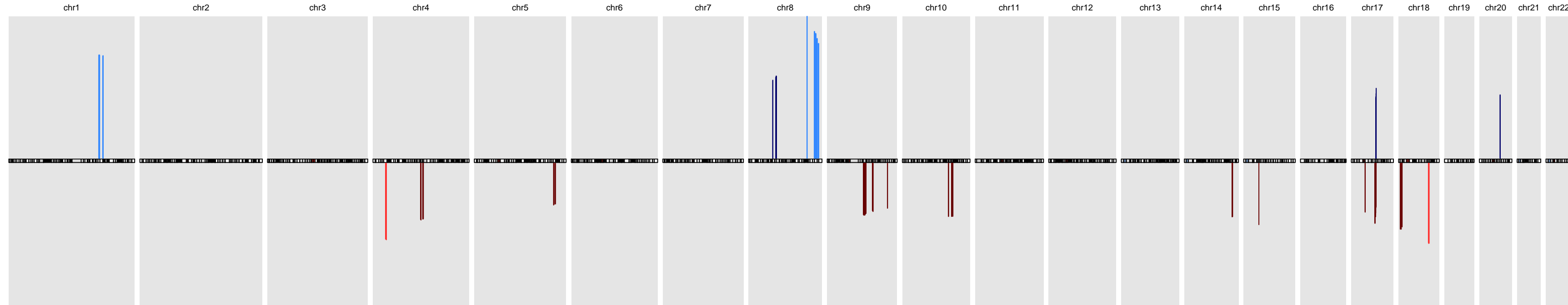

Supplement: Supplementary file 3 [file Data_Sheet_3.ZIP › signatures/Breast/Infiltrating duct carcinoma/sigGenes_full.pdf]

# Breast Intraductal carcinoma: 8500/2

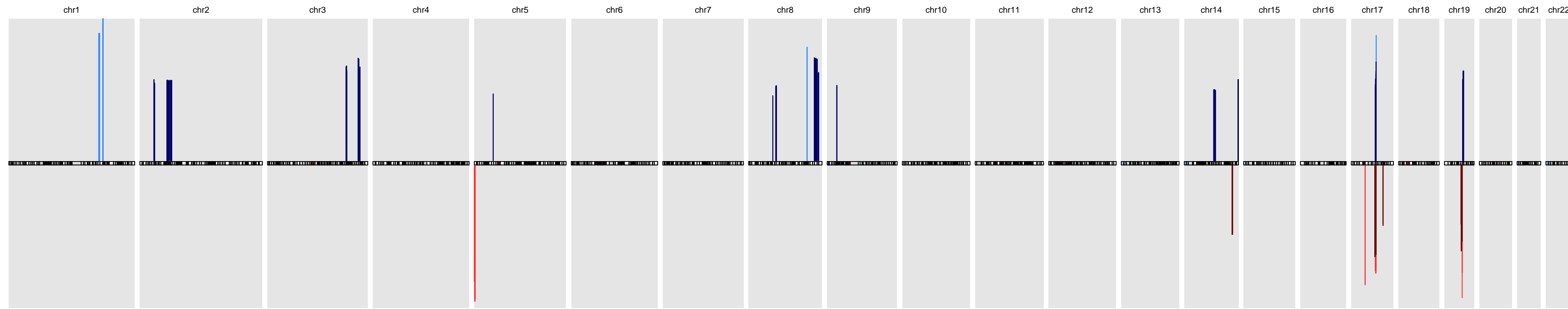

Supplement: Supplementary file 3 [file Data_Sheet_3.ZIP › signatures/Breast/Intraductal carcinoma/sigGenes_full.pdf]

# Breast Lobular carcinoma: 8520/3

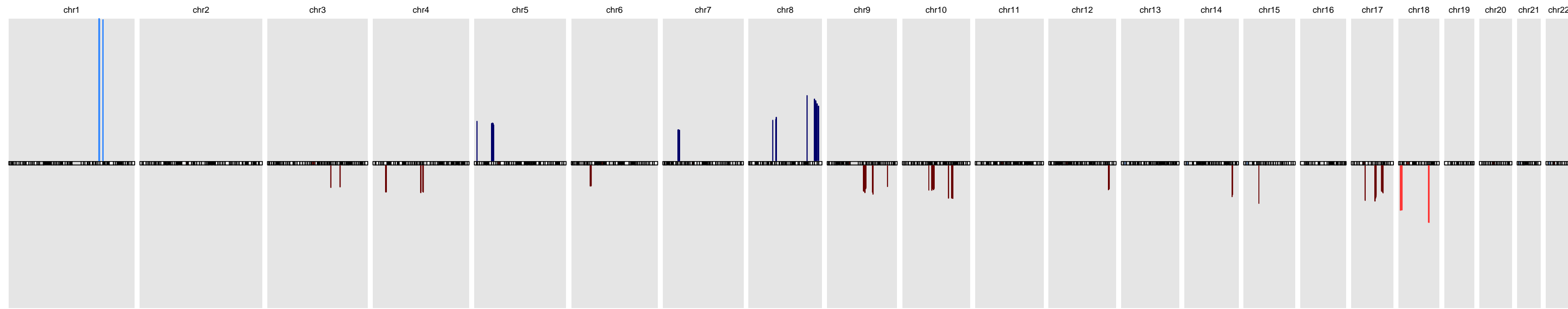

Supplement: Supplementary file 3 [file Data_Sheet_3.ZIP › signatures/Breast/Lobular carcinoma/sigGenes_full.pdf]

# Kidney Renal cell carcinoma 8312/3

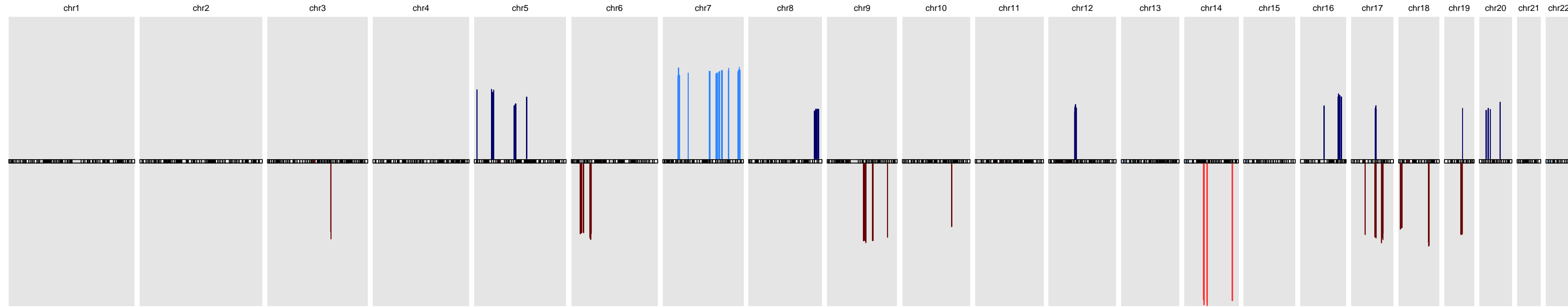

Supplement: Supplementary file 3 [file Data_Sheet_3.ZIP › signatures/Kidney/Renal cell carcinoma/sigGenes_full.pdf]

# Kidney Clear cell adenocarcinoma 8310/3

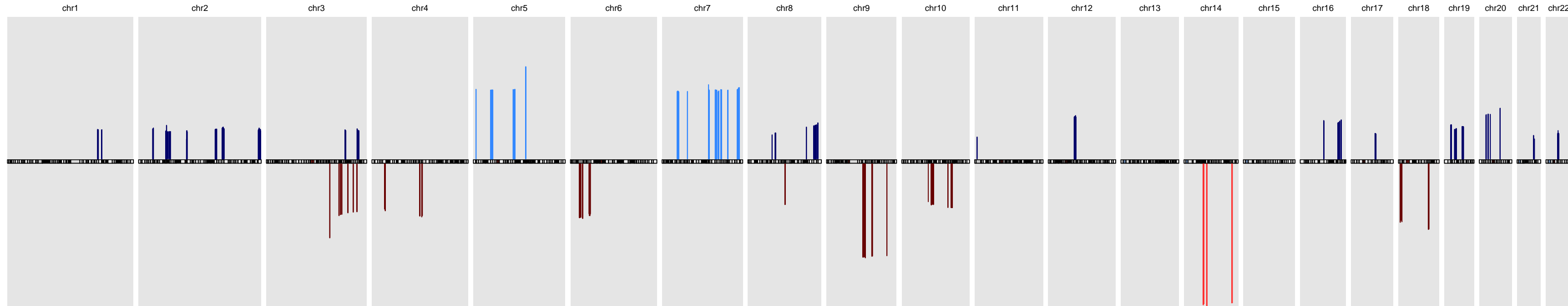

Supplement: Supplementary file 3 [file Data_Sheet_3.ZIP › signatures/Kidney/Clear cell adenocarcinoma/sigGenes_full.pdf]

# Colon Adenoma: 8140/0

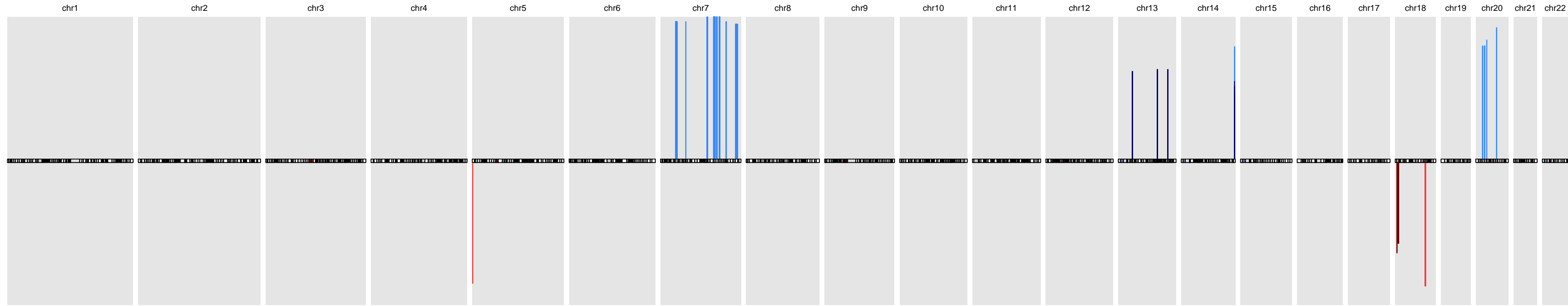

Supplement: Supplementary file 3 [file Data_Sheet_3.ZIP › signatures/Colon/Adenoma/sigGenes_full.pdf]

# Colon Adenocarcinoma: 8140/3

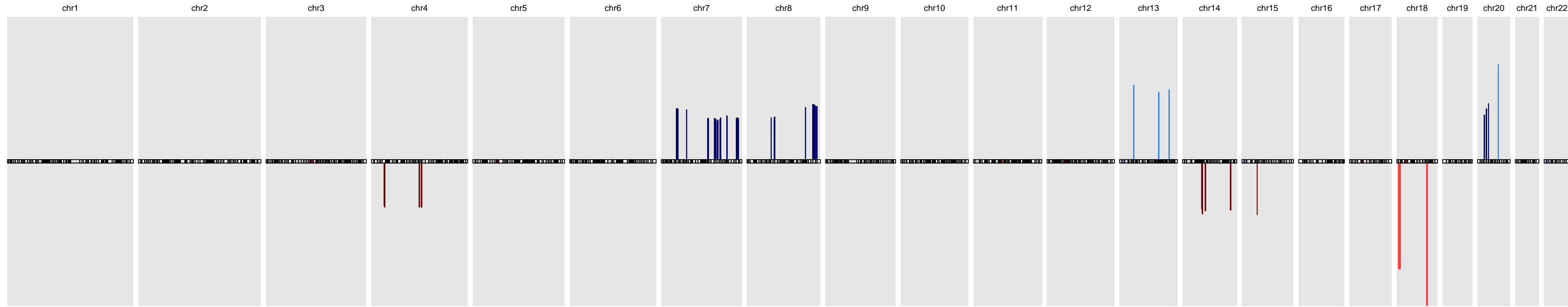

Supplement: Supplementary file 3 [file Data_Sheet_3.ZIP › signatures/Colon/Adenocarcinoma/sigGenes_full.pdf]

# Colon Adenocarcinoma intestinal type: 8144/3

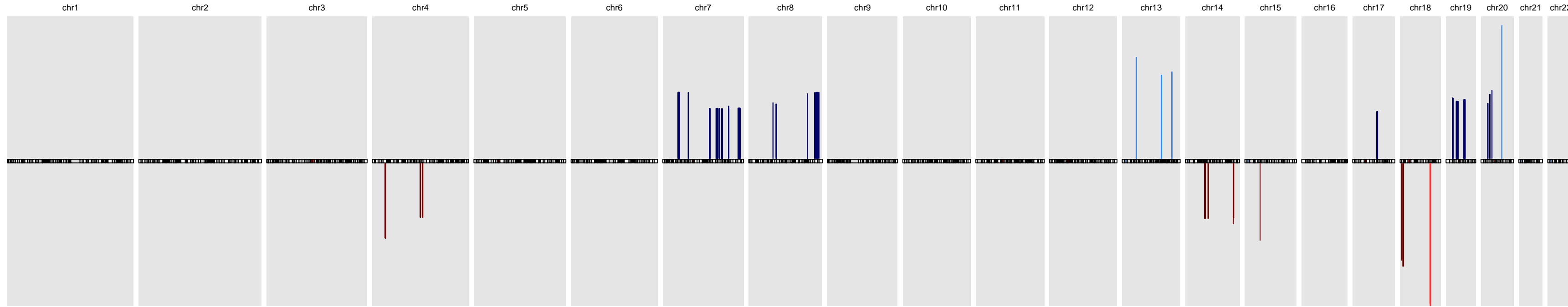

Supplement: Supplementary file 3 [file Data_Sheet_3.ZIP › signatures/Colon/Adenocarcinoma intestinal type/sigGenes_full.pdf]

# Colon Mucinous adenocarcinoma: 8480/3

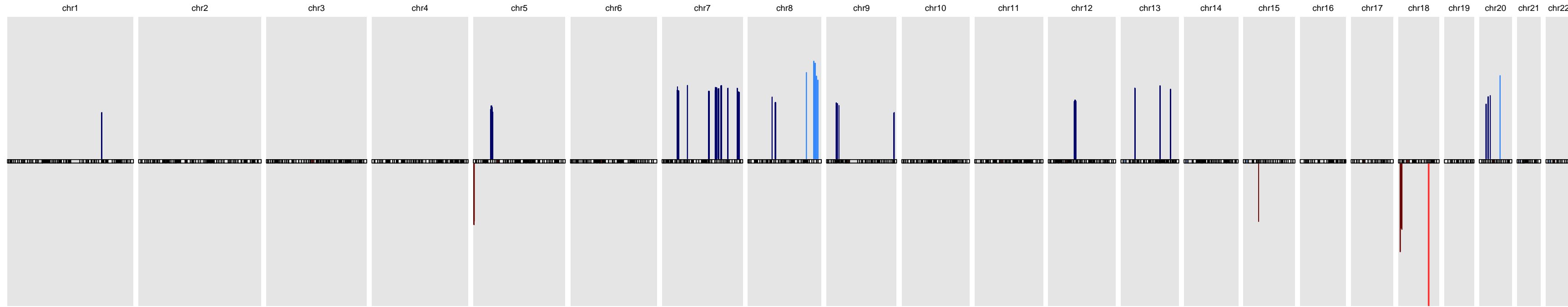

Supplement: Supplementary file 3 [file Data_Sheet_3.ZIP › signatures/Colon/Mucinous adenocarcinoma/sigGenes_full.pdf]

# Cerebellum Medulloblastoma 9470/3, 9471/3, 9474/3

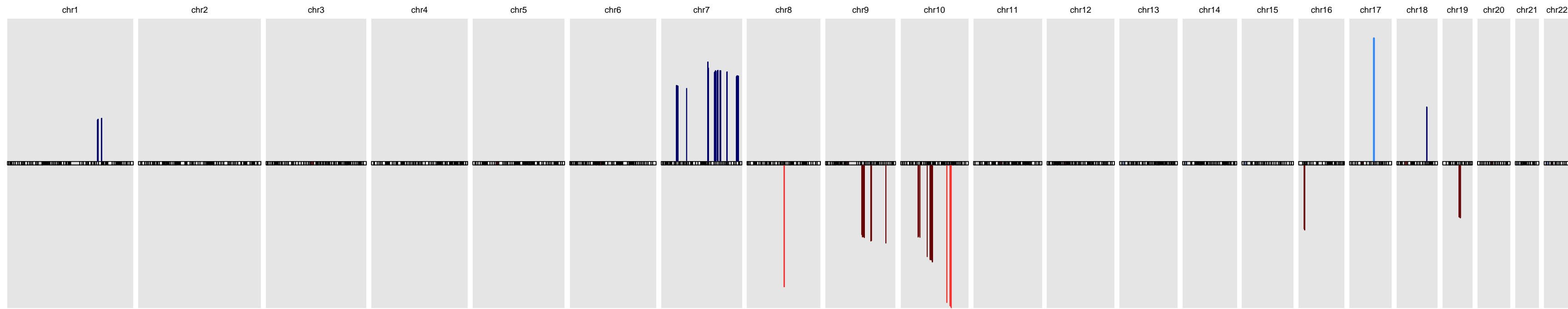

Supplement: Supplementary file 3 [file Data_Sheet_3.ZIP › signatures/Cerebellum/Medulloblastoma/sigGenes_full.pdf]

# Stomach Gastrointestinal stromal sarcoma 8936/3

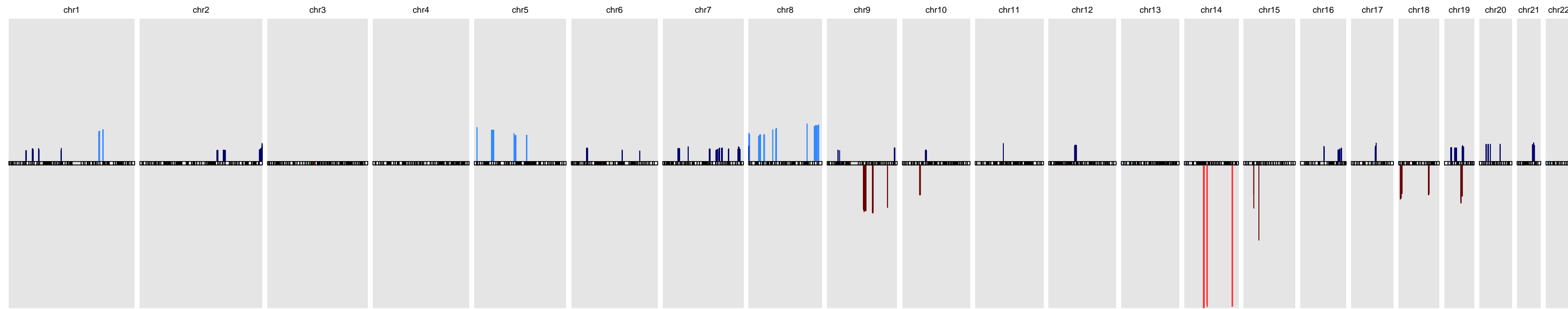

Supplement: Supplementary file 3 [file Data_Sheet_3.ZIP › signatures/Stomach/Gastrointestinal stromal sarcoma/sigGenes_full.pdf]

# Stomach Adenocarcinoma 8140/3

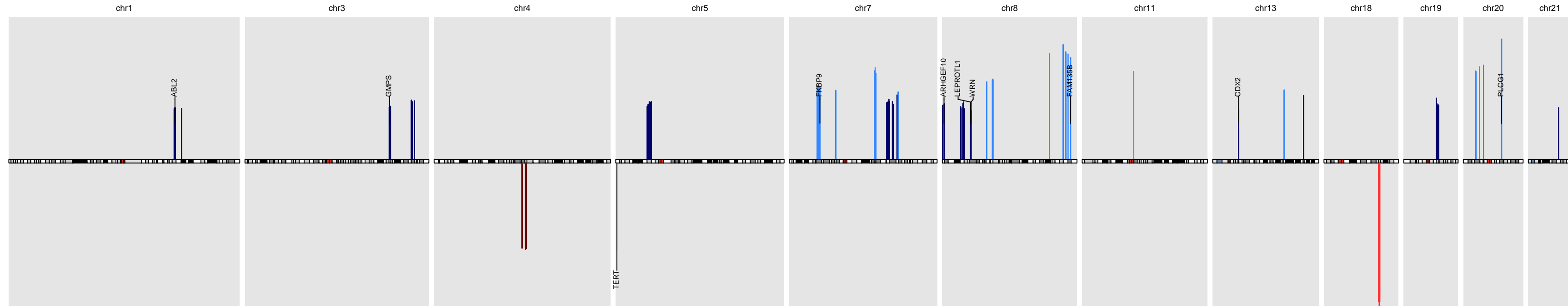

Supplement: Supplementary file 3 [file Data_Sheet_3.ZIP › signatures/Stomach/Adenocarcinoma/sigGenes.pdf]

# Stomach Carcinoma diffuse type 8145/3

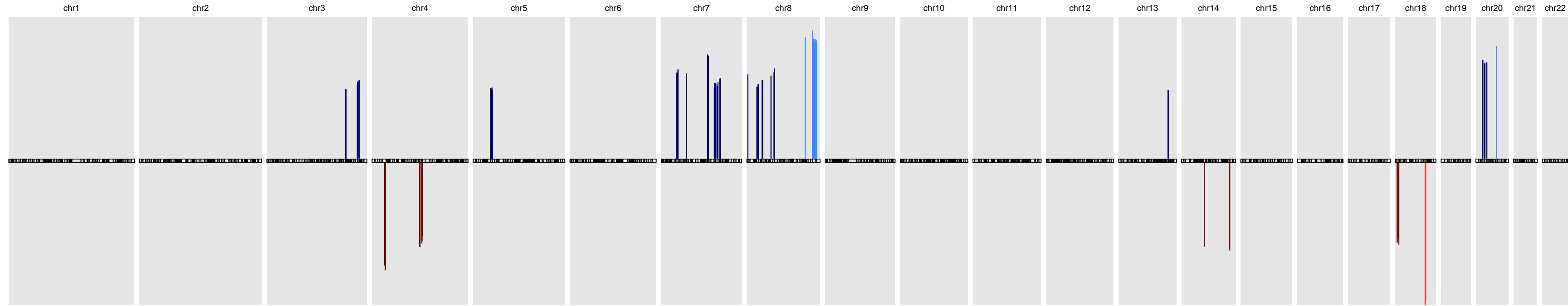

Supplement: Supplementary file 3 [file Data_Sheet_3.ZIP › signatures/Stomach/Carcinoma diffuse type/sigGenes_full.pdf]

# Stomach Adenocarcinoma intestinal type 8144/3

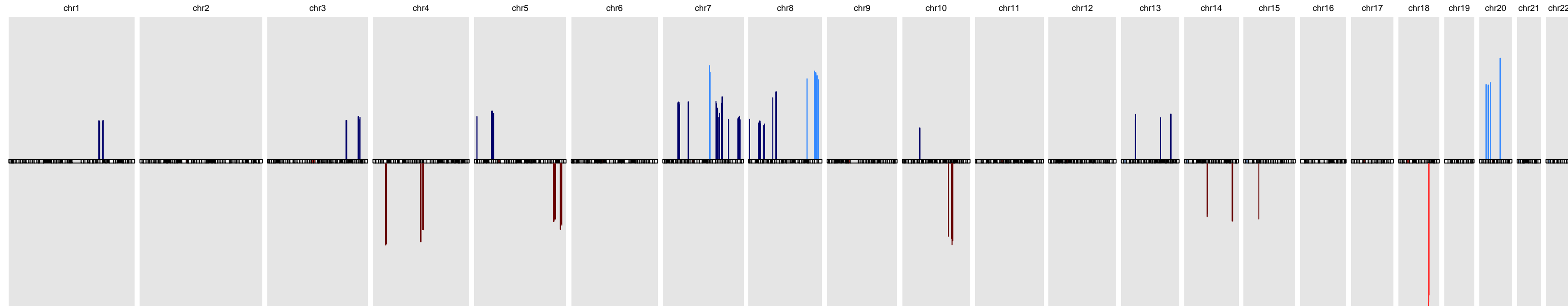

Supplement: Supplementary file 3 [file Data_Sheet_3.ZIP › signatures/Stomach/Adenocarcinoma intestinal type/sigGenes_full.pdf]

# Stomach Tubular adenocarcinoma 8211/3

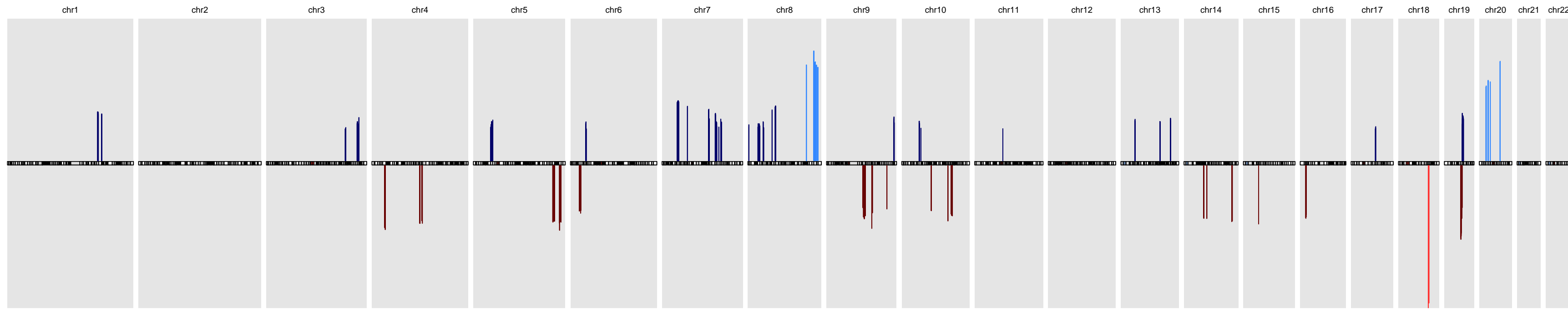

Supplement: Supplementary file 3 [file Data_Sheet_3.ZIP › signatures/Stomach/Tubular adenocarcinoma/sigGenes_full.pdf]

# Brain Astrocytoma: 9400/3, 9401/3

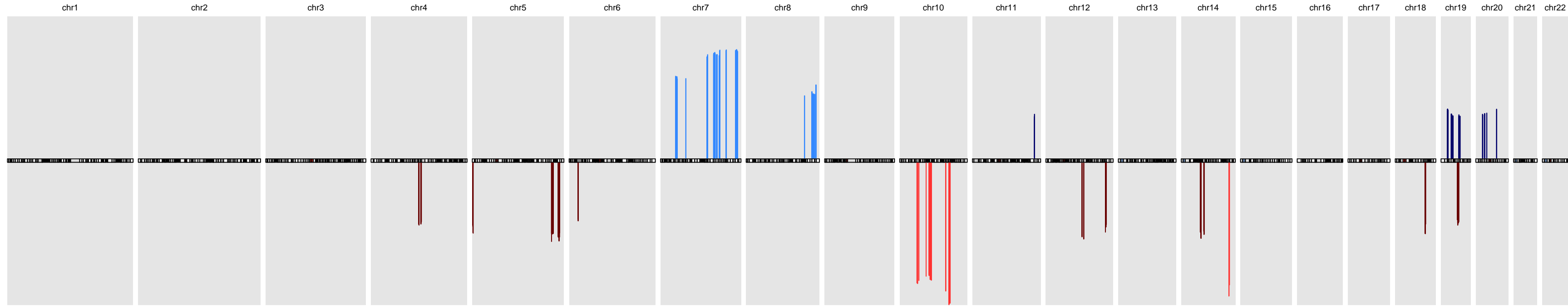

Supplement: Supplementary file 3 [file Data_Sheet_3.ZIP › signatures/Brain/Astrocytoma/sigGenes_full.pdf]

# Brain Glioma: 9380/3, 9440/3

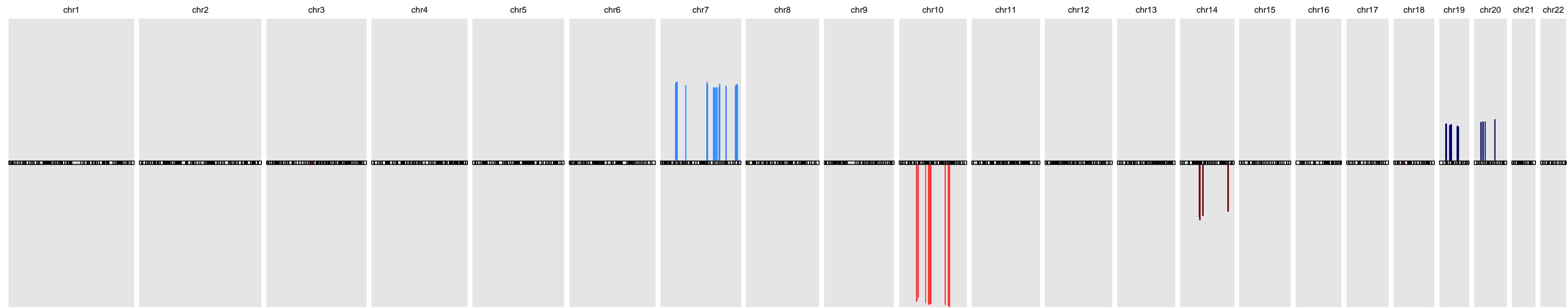

Supplement: Supplementary file 3 [file Data_Sheet_3.ZIP › signatures/Brain/Glioma/sigGenes_full.pdf]

Brain Oligodendroglioma: 9450/3, 9451/3

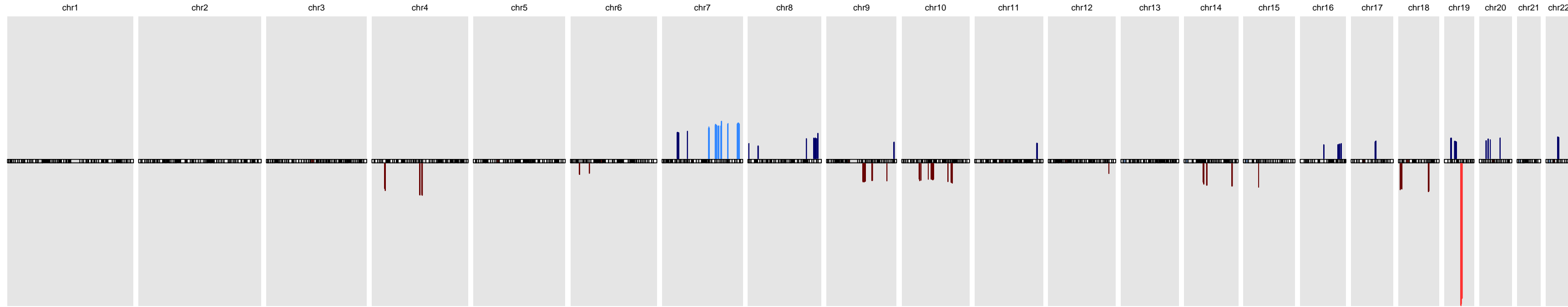

Supplement: Supplementary file 3 [file Data_Sheet_3.ZIP › signatures/Brain/Oligodendroglioma/sigGenes_full.pdf]

# Brain Mixed glioma: 9382/3

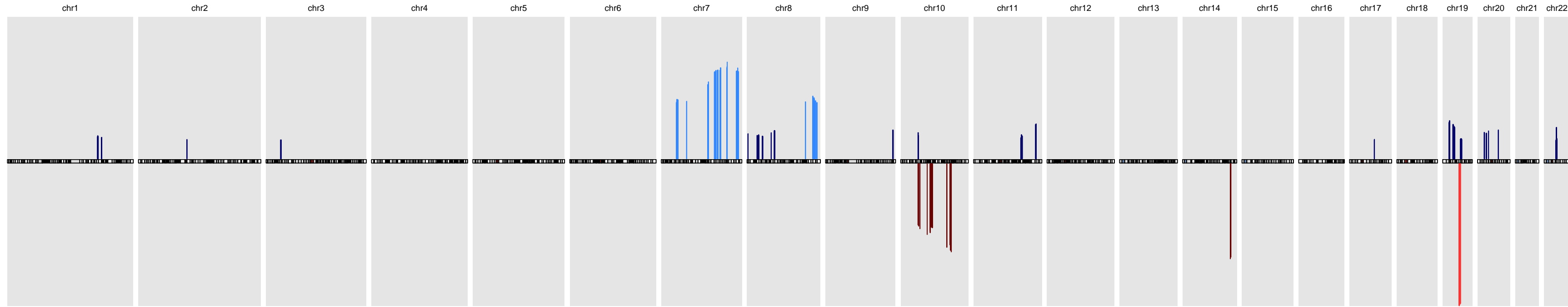

Supplement: Supplementary file 3 [file Data_Sheet_3.ZIP › signatures/Brain/Mixed glioma/sigGenes_full.pdf]

# Liver Hepatocellular carcinoma 8170/3

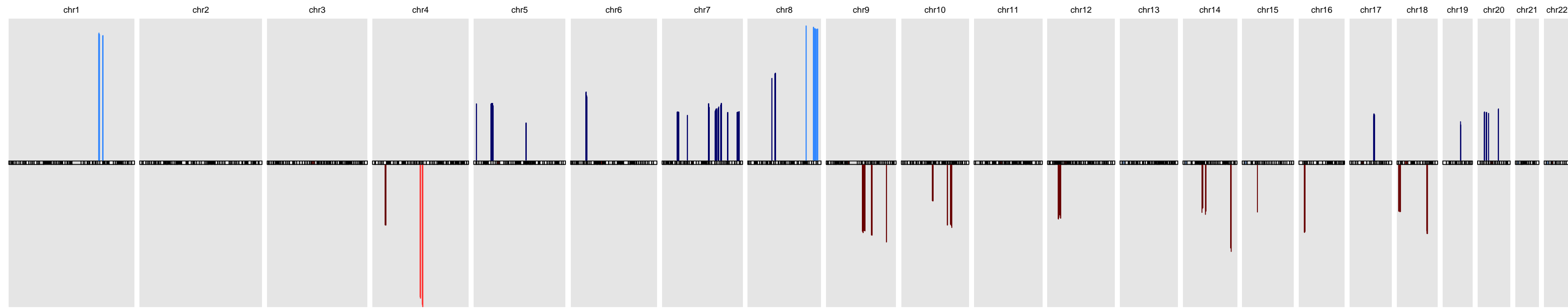

Supplement: Supplementary file 3 [file Data_Sheet_3.ZIP › signatures/Liver/Hepatocellular carcinoma/sigGenes_full.pdf]

# Ovary Carcinoma: 8010/3, 8441/3, 8442/1

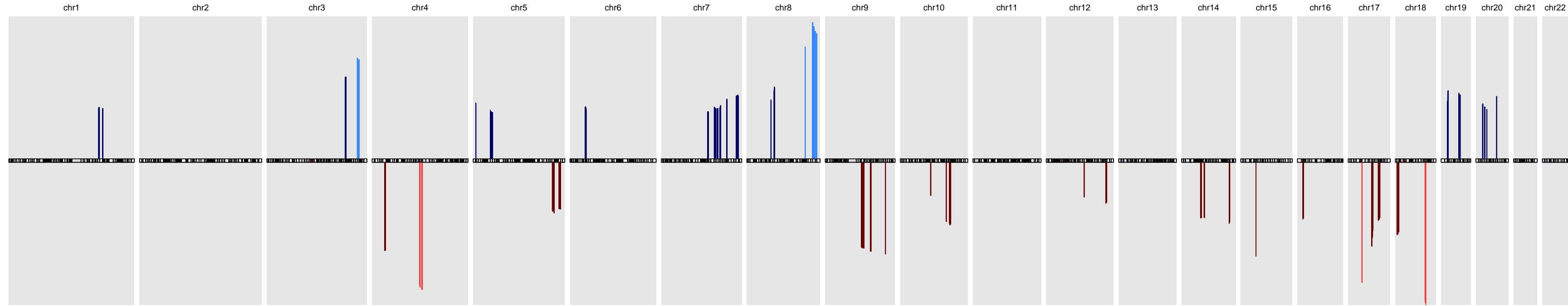

Supplement: Supplementary file 3 [file Data_Sheet_3.ZIP › signatures/Ovary/Carcinoma/sigGenes_full.pdf]

# Ovary Adenocarcinoma: 8140/3, 8310/3, 8380/3

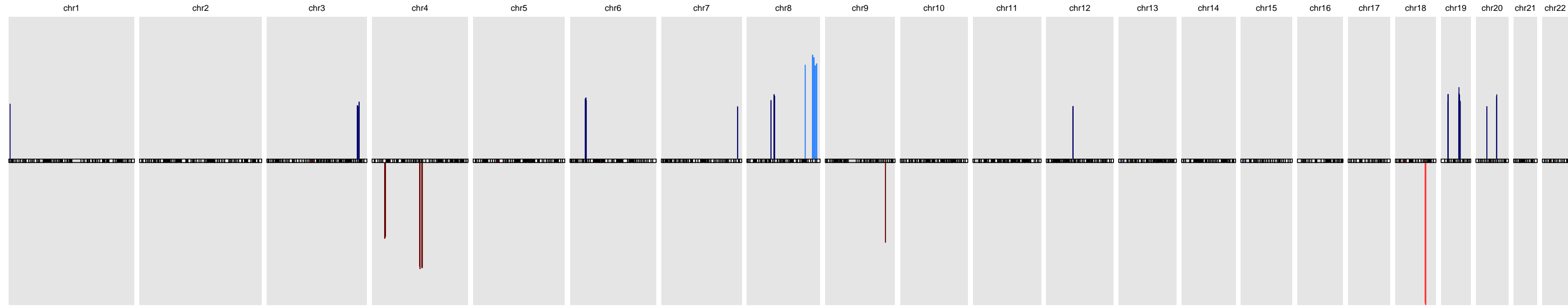

Supplement: Supplementary file 3 [file Data_Sheet_3.ZIP › signatures/Ovary/Adenocarcinoma/sigGenes_full.pdf]

# Ovary Mucinous cystadenoma: 8470/0, 8480/0

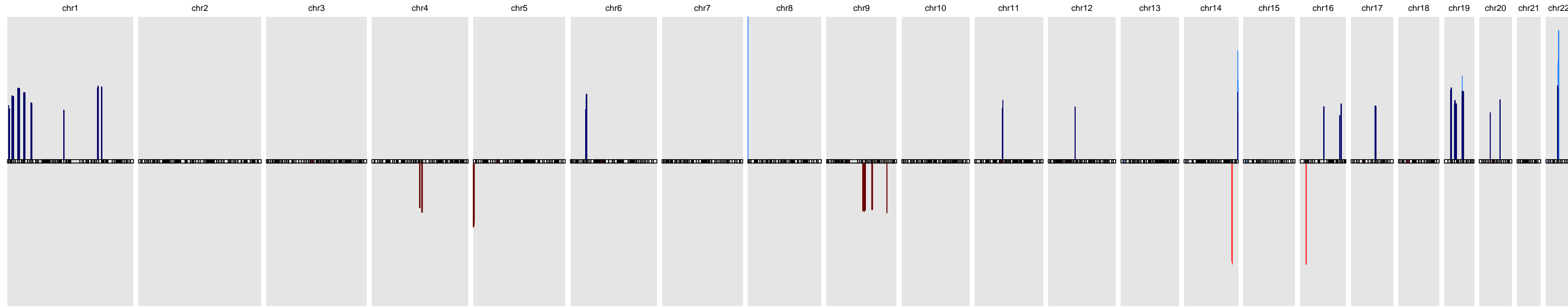

Supplement: Supplementary file 3 [file Data_Sheet_3.ZIP › signatures/Ovary/Mucinous cystadenoma/sigGenes_full.pdf]
