# Supplementary material for: Signatures of Discriminative Copy Number Aberrations in 31 Cancer Subtypes
Source: Front Genet. 2021 May 13;12:654887. doi: 10.3389/fgene.2021.654887 (PMC8155688; doi:10.3389/fgene.2021.654887)
Supplement: Supplementary file 4 [file Data_Sheet_4.PDF]

Merged based on signatures, ■ is left out (less than 50).

Tissue	Analyzed subtypes	Annotated subtypes	IDCO morphology	Samples
Brain	Glioma, malignant	Glioma, malignant	9380/3	159
		Glioblastoma	9440/3	1646
	Astrocytoma	Astrocytoma	9400/3	160
		Astrocytoma, anaplastic	9401/3	179
	Oligodendroglioma	Oligodendroglioma	9450/3	221
		Oligodendroglioma, anaplastic	9451/3	105
	Primitive neuroectodermal tumor		9473/3	82
	Mixed glioma (Anaplastic oligoastrocytoma, Oligoastrocytoma)		9382/3	162
		Ependymoma	9391/3	49
Breast	Intraductal carcinoma, noninfiltrating, NOS		8500/2	82
	Infiltrating duct carcinoma, NOS		8500/3	5657
	Lobular carcinoma, NOS		8520/3	201
		Infiltrating duct and lobular carcinoma	8522/3	28
		Infiltrating duct mixed with other types of carcinoma	8523/3	14
		Inflammatory carcinoma	8530/3	19
		Metaplastic carcinoma, NOS	8575/3	16
		Pleomorphic carcinoma	8022/3	14
		Mucinous adenocarcinoma	8480/3	15
		Intraductal micropapillary carcinoma (8507/2)	8507/3	43
Cerebellum	Medulloblastoma, NOS	Medulloblastoma, NOS	9470/3	1573
		Desmoplastic nodular medulloblastoma	9471/3	72
		Large cell medulloblastoma	9474/3	37
Colon	Adenoma, NOS		8140/0	63
	Adenocarcinoma, NOS		8140/3	1643
	Adenocarcinoma, intestinal type		8144/3	53
	Mucinous adenocarcinoma		8480/3	62
Kidney	Clear cell adenocarcinoma, NOS		8310/3	930
	Renal cell carcinoma, NOS		8312/3	323

Tissue	Analyzed subtypes	Annotated subtypes	IDCO morphology	Samples
		Renal cell carcinoma, chromophobe type	8317/3	15
		Oxyphilic adenoma	8290/0	17
Liver	Hepatocellular carcinoma		8170/3	371
Lung	Carcinoma, NOS	Carcinoma, NOS	8010/3	84
		Large cell carcinoma, NOS	8012/3	54
	Adenocarcinoma	Adenocarcinoma	8140/3	1103
		Adenocarcinoma with mixed subtypes	8255/3	109
	Small cell carcinoma, NOS		8041/3	155
	Non-small cell carcinoma		8046/3	1725
	Squamous cell carcinoma, NOS		8070/3	518
		Squamous cell carcinoma, uncertain	8070/1	12
	Bronchiolo-alveolar adenocarcinomas	Bronchiolo-alveolar adenocarcinoma, NOS	8250/3	24
		Bronchiolo-alveolar carcinoma, non-mucinous	8252/3	19
		Bronchiolo-alveolar carcinoma, mixed mucinous and non-mucinous	8254/3	18
		Papillary adenocarcinoma, NOS	8260/3	29
		Mucinous adenocarcinoma	8480/3	14
		Acinar cell carcinoma	8550/3	21
		Adenosquamous carcinoma	8560/3	16
Ovary	Carcinoma	Carcinoma	8010/3	761
		Serous carcinoma	8441/3	964
		Serous tumor	8442/1	45
	Mucinous cystadenoma	Mucinous cystadenoma	8470/0	57
		Mucinous adenoma	8480/0	60
	Adenocarcinoma		8140/3	111
		Clear Cell Adenocarcinoma	8310/3	24
		Endometrioid adenocarcinoma	8380/3	11
Prostate	Adenocarcinoma		8140/3	916
		Carcinoma, NOS	8010/3	16
	Malignant melanoma, NOS	Malignant melanoma, NOS	8720/3	1030
		Nodular melanoma	8721/3	22
		Amelanotic melanoma	8730/3	16

Tissue	Analyzed subtypes	Annotated subtypes	IDCO morphology	Samples
Skin		Pigmented dermatofibrosarcoma protuberans (Bednar tumor)	8833/3	11
		Mycosis fungoides (Pagetoid reticulosis)	9700/3	32
		Epidermoid carcinoma (Squamous cell carcinoma), NOS	8070/3	18
		Keratinizing (Squamous cell carcinoma)	8071/0	11
Stomach	Adenocarcinoma		8140/3	763
	Gastrointestinal stromal sarcoma		8936/3	175
	Adenocarcinoma, intestinal type		8144/3	83
	Carcinoma, diffuse type		8145/3	57
	Tubular adenocarcinoma		8211/3	82
		Mucinous adenocarcinoma	8480/3	21
		Signet ring cell carcinoma	8490/3	16
		Carcinoma	8010/3	19
		Adenoma, NOS	8140/0	17
		Adenocarcinoma in situ, NOS	8140/2	19
